# Evaluation of *Arthrobacter aurescens* Strain TC1 as Bioaugmentation Bacterium in Soils Contaminated with the Herbicidal Substance Terbuthylazine

**DOI:** 10.1371/journal.pone.0144978

**Published:** 2015-12-14

**Authors:** Vera P. Silva, Matilde Moreira-Santos, Carla Mateus, Tânia Teixeira, Rui Ribeiro, Cristina A. Viegas

**Affiliations:** 1 iBB-Institute for Bioengineering and Biosciences, Instituto Superior Técnico (IST), Universidade de Lisboa (UL), Lisboa, Portugal; 2 Department of Bioengineering, IST, UL, Lisboa, Portugal; 3 CFE-Centre for Functional Ecology, Department of Life Sciences, University of Coimbra, Coimbra, Portugal; Yeungnam University, REPUBLIC OF KOREA

## Abstract

In the last years the chloro-*s*-triazine active substance terbuthylazine has been increasingly used as an herbicide and may leave residues in the environment which can be of concern. The present study aimed at developing a bioaugmentation tool based on the soil bacterium *Arthrobacter aurescens* strain TC1 for the remediation of terbuthylazine contaminated soils and at examining its efficacy for both soil and aquatic compartments. First, the feasibility of growing the bioaugmentation bacterium inocula on simple sole nitrogen sources (ammonium and nitrate) instead of atrazine, while still maintaining its efficiency to biodegrade terbuthylazine was shown. In sequence, the successful and quick (3 days) bioremediation efficacy of ammonium-grown *A*. *aurescens* TC1 cells was proven in a natural soil freshly spiked or four-months aged with commercial terbuthylazine at a dose 10× higher than the recommended in corn cultivation, to mimic spill situations. Ecotoxicity assessment of the soil eluates towards a freshwater microalga supported the effectiveness of the bioaugmentation tool. Obtained results highlight the potential to decontaminate soil while minimizing terbuthylazine from reaching aquatic compartments via the soil-water pathway. The usefulness of this bioaugmentation tool to provide rapid environment decontamination is particularly relevant in the event of accidental high herbicide contamination. Its limitations and advantages are discussed.

## Introduction

Terbuthylazine (TBA; 2-*tert*-butylamino-4-chloro-6-ethylamino-1, 3, 5-triazine) is used worldwide to control broad-leaved and grassy weeds in agriculture and forestry situations as well as slime-forming algae, fungi and bacteria in non-agricultural situations [1–3]. This active substance is being increasingly used in the EU since atrazine (ATZ) ban in 2008 [1, 4]. Agricultural and disposal practices in accordance with regulatory guidelines should ensure no TBA risk for the environment or human health. In recent years, however, apprehension over possible risks of TBA and its chlometabolites (e.g., desethylterbuthylazine, DET, among others, which can be formed from the *N*-dealkylation of the *s*-triazine-ring lateral amines by soil microorganisms [1, 3]) have been raised. Concerns have been mainly related with: (i) detection of TBA and DET in aquatic compartments at levels above the maximum admissible concentration in drinking and ground waters (e.g., 0.1 μg L^-1^ in the EU) [1, 5, 6]; (ii) moderate to high risk of these compounds for non-target aquatic organisms [1, 2, 7, 8]; (iii) TBA and DET potential to provoke endocrine disruption in mammalian cells [9, 10]; and (iv) TBA ability to potentiate toxic effects of other pesticides in zebrafish [8]. Worrisome environmental contamination by TBA may occur due to aerial drift, accidental spills and deficient storage at dealerships and mix-load sites [5, 11, 12]. Upon soil irrigation and/or intensive rainfalls TBA and its dehalkylated chlorometabolite(s) can migrate from soils to nearby surface and ground waters, by runoff and/or leaching, where they can be highly persistent [2, 6, 12].

To mitigate environmental risk of *s*-triazine herbicides, bioremediation strategies based on bioaugmentation of soil, sediments or waters with microorganisms able to convert these compounds into less toxic products have been proposed [5, 11, 13–18]. Such studies have involved mostly ATZ, and, as far as we are aware, only few focused on the biodegradation of the less bioavailable TBA in soil [15, 18]. In particular, contrary to ATZ [13, 14, 16, 17], the examination of both the bioremediation efficacy in soils and ability to minimize herbicide dispersion via the soil-water pathway at TBA concentrations relevant to spill situations in agricultural soils (e.g., 10× the recommended dose in crop growing, or higher [13, 14]) is limited. The soil bacterium *Arthrobacter aurescens* strain TC1, isolated at a roadside herbicide spill site in the US, is able to use ATZ and many other chloro-*s*-triazine herbicides including TBA, as nitrogen and/or carbon sources [19, 20]. *A*. *aurescens* TC1 was reported to biodegrade ATZ much more efficiently than most of the described *s*-triazine-degrading microorganisms (including the better-studied *Pseudomonas* sp. ADP [13, 14, 16, 18, 20]), and the same can be anticipated for TBA [20, 21]. Triazine catabolism in *A*. *aurescens* TC1 is well established; it occurs via hydrolytic displacement of the chlorine and amine substituents of the *s*-triazine ring mediated by the following three enzymes: TrzN (catalyzes the initial dechlorination step to produce a hydroxy-*s*-triazine metabolite), AtzB and AtzC (both catalyze the sequential deamination of the lateral *N*-alkylamines) [20]. This catabolic pathway funnels into cyanuric acid, which accumulates stoichiometrically (1:1) and is no further mineralized [19, 20]. The performance of these reactions is environmentally relevant because hidroxy-*s*-triazines and cyanuric acid pose lower risks to soil and aquatic organisms compared with the parent compounds [1, 16].

In this context, the aims of the present work were (i) to develop a bioaugmentation tool for bioremediation of soils contaminated with TBA concentrations relevant to accidental spill scenarios, focusing on the optimization of *A*. *aurescens* TC1 inocula, and (ii) to examine the efficacy of the bioaugmentation tool in TBA-contaminated soil microcosms, with emphasis on the need to also rapidly reduce the dispersion of herbicide-associated toxicity to aquatic compartments. With respect to the former, and while intending to verify whether the incorporation of ATZ in the *A*. *aurescens* TC1 growth medium [19, 22] could be avoided, the growth of *A*. *aurescens* TC1 inocula on four different sole nitrogen sources (ammonium, urea, nitrate and ATZ) was compared as well as the ability of these cultures to effectively biodegrade TBA. Our approach was motivated by the fact that studies on soil bioaugmentation with chloro-*s*-triazine-degrading bacteria have mostly involved inoculum cultures grown under selective pressure with ATZ as sole nitrogen source [11, 13, 14, 17–19]. It is a fact that such procedure overcomes problems due to the instability of *s*-triazine catabolic plasmids [20, 23] and the nitrogen-dependent control of chloro-*s*-triazines degradation found in a number of chloro-*s*-triazine degrading bacteria (reviewed in [24]). However, in our view, growing bacterial cultures in media containing xenobiotics (e.g., ATZ) for the bioaugmentation of contaminated environments is a contradiction-in-terms. Moreover, it may constitute a drawback of the bioremediation process, as it most likely leads to toxic chemical residues that for safety purposes should be disposed. Upon confirmation of ammonium as the most promising nitrogen source for *A*. *aurescens* TC1growth, the second study aim intended to evaluate the usefulness of ammonium-grown *A*. *aurescens* TC1 inoculum for the bioremediation of soil microcosms freshly- or aged-spiked with a commercial TBA formulation (to mimic accidental spills). To further assess the ability of the *A*. *aurescens* TC1 bioaugmentation tool in providing complete and rapid environment decontamination, i.e., of soils while simultaneously minimizing the spread of herbicide-associated toxicity to aquatic compartments, the ecotoxicity testing of soil eluates using a standard freshwater microalga highly sensitive to *s*-triazine herbicides [13, 14] was performed.

## Materials and Methods

### Chemicals

Terbuthylazine (TBA), atrazine (ATZ) (both Pestanal, purity 99.1%) and urea (≥ 95%) were purchased from Sigma-Aldrich (Seelze, Germany), (NH_4_)_2_SO_4_ from Panreac (Barcelona, Spain) and NaNO_3_ from Merck (Darmstadt, Germany). The formulated herbicide Terbutilazina-Sapec (concentrate suspension of 0.5 kg active substance L^-1^; recommended field dose (RD) for weed control in corn plantations of 1.5 L ha^-1^) was purchased from Sapec—Agro (Setúbal, Portugal).

### Microorganism and growth on different nitrogen sources

The *Arthrobacter aurescens* strain TC1 (ATCC BAA-1386, kindly offered by L.P. Wackett, University of Minnesota, US) [19], was used. Liquid TC1 base growth medium (pH 7.0±0.1) contained glucose (1.8 g L^-1^) and trisodium citrate (2 g L^-1^) as carbon sources [22] plus vitamins (final concentrations in mg L^-1^: thiamine-HCl 0.1; biotin 0.04; folic acid 0.04; niacinamide 0.2; pyridoxine-HCl 0.2) and salts (final concentrations in mg L^-1^: KH_2_PO_4_ 9120; EDTA 50; MgSO_4_.7H_2_O 400; ZnSO_4_ 222; FeSO_4_ 100; MnSO_4_.5H_2_O 31; CuSO_4_.5H_2_O 8; Na_2_B_4_O_7_.10H_2_O 3.6; H_2_SO_4_ 4.9). To evaluate ammonium, urea or nitrate as sole nitrogen sources, TC1 base medium was supplemented with (NH_4_)_2_SO_4_, urea or NaNO_3_ respectively, at final soluble concentrations providing either 2.8 or 10 mM nitrogen. ATZ as sole nitrogen source was tested only at the saturating concentration of 300 mg L^-1^ (from a stock solution at 16200 mg L^-1^ in methanol), corresponding to approximately 2.8 mM nitrogen usable by *A*. *aurescens* TC1 [20]. Although the concentration of soluble ATZ in the growth medium cannot be precise due to the ATZ low water solubility (35 mg L^-1^ at 20°C) [2], a continuous ATZ supply to the cells keeping pace with its continuous biodegradation can be assumed [19]. Unless otherwise indicated, bacterial cultures were grown at 30°C in an orbital shaker (250 rpm).

In the growth experiments, pre-inoculum cultures grown overnight to late-exponential phase in TC1 base medium supplemented with the required sole nitrogen source were used to inoculate fresh medium with the same composition (40 ml in 100-ml Erlenmeyer flasks) to give an initial culture optical density at 640 nm (OD_640_) of 0.1, corresponding to approximately 2.4 × 10^7^ colony forming units (cfu) ml^-1^. Growth curves were monitored during 30 h of incubation (i.e., up to the stationary phase of growth) by measuring both the OD_640_ and the concentration of viable cells. The latter was assessed by counting cfu in 0.1 ml of culture serial dilutions spread plated onto agarized Lennox Broth (LB) upon 48 h incubation at 30°C. The specific growth rate (in h^-1^) was calculated by least-square fitting to the linear part of the semi-logarithmic plots of OD_640_ or cfu ml^-1^ versus time (in h); because specific growth rates calculated based on OD_640_ or cfu ml^-1^ were concordant only the former are shown in the Results section. Two (ATZ) to three (ammonium, urea, or nitrate) biological replicates were set up per treatment.

### Herbicide degradation by cells grown in different nitrogen sources

To address whether cells grown on 2.8 mM of nitrogen from ammonium, urea or nitrate, compared to ATZ, were able to biodegrade TBA (and also ATZ, for comparison purposes), a resting cell degradation assay [25] was adapted. In the present study, mid-exponential *A*. *aurescens* TC1 cultures (OD_640_ ~ 0.4) grown on each nitrogen source (as described above) were harvested by centrifugation (5 min, 4°C, 10000×*g*) and washed twice in sterile saline solution. To start the herbicide degradation assay, cells were suspended in 40 ml of thermostatized (30°C) sterile phosphate-salt buffer (10 mM sodium phosphate, 0.1 mM MgSO_4_, 0.1 mM ZnSO_4_; pH 7.0±0.2) supplemented with ~0.05 mM of TBA or ATZ to give an OD_640_ of 0.10 ± 0.01 (corresponding to 2.5±0.8 × 10^7^ cfu ml^-1^). The obtained cell suspensions were immediately incubated at 30°C in an orbital shaker (250 rpm). Controls without cells or with cells killed by boiling (5 min) were also performed. To obtain the time-course curves of TBA or ATZ removal by the bacterial cells, samples (4 ml; duplicated) were withdrawn at adequate time intervals (from zero up to 24 or 5 h for TBA or ATZ, respectively) and centrifuged immediately (5 min, 4°C, 10000×*g*); supernatants were collected and stored at -20°C until analysis. The herbicide concentrations in the supernatants were determined by reversed-phase high performance liquid chromatography (HPLC) in a Hitachi L-2300 (La Chrom Elite, San Jose, CA, US) equipped with a LiChro CART 250–4 RP-18 column and a UV detector (223 nm), using a gradient mobile phase of water/acetonitrile with a flow rate of 1 ml min^-1^, at 40°C. ATZ and TBA peaks were detected at 35 and 46 min, respectively, and the respective concentrations determined from each peak area based on calibration curves of herbicide standard solutions (linear up to at least 0.05 mM). TBA or ATZ degradation rates (expressed as mmol herbicide h^-1^) were estimated as the slope of the straight line tangent to the plots of concentration versus time (in h), in the time-periods between zero and 4 or 2 h, respectively. To calculate the respective specific degradation rates, the calculated slopes were divided by the number of *A*. *aurescens* TC1 viable cells (as cfu) present at the beginning of each assay (cfu assessed as described above). For each treatment, data for biodegradation curves were obtained from two (ATZ, urea, nitrate) or five (ammonium) replicates.

### Bioremediation experiments in soil microcosms

A natural sandy loam soil representative of a corn production field from Central Portugal (collected at Escola Superior Agrária de Coimbra, Coimbra, Portugal) was used; its main characteristics are described elsewhere [26]. Soil was sieved (5 mm) and stored at field moisture content in plastic bags, at 4°C, until used. Water content (13.8±0.4%), water holding capacity (32.2±3.6%) and pH (7.0±0.2) were analysed as described in Lima et al. [26]. Soil microcosms containing 150 g dry weight (dw) of soil were set up in glass cylinders as previously described [13, 26]. The natural soil was spiked with an aqueous suspension of the commercial formulation Terbutilazina-Sapec (2.8 ml per 100 g of soil dw) to achieve a TBA concentration of 10 mg kg^-1^ dw of soil, i.e., equal to 10×RD for corn cultivation (assuming 1×RD equivalent to 0.75 kg active substance ha^-1^ distributed into a 5 × 5 cm soil column with an average soil density of 1.5 g cm^-3^ [13, 26]); 10×RD intended to represent worst-case situations of soil herbicide contamination associated with, for example, accidental spills or concentration hotspots in land and dealership mix-load or disposal sites [11, 13, 14, 16]. Soil was either freshly spiked or four-months aged (stored at 4°C [18]) upon herbicide spiking. In both cases, the TBA-contaminated soil was bioaugmented by distributing adequate volumes of an inoculum suspension (3.1 ± 0.4 × 10^10^ cfu of *A*. *aurescens* TC1 ml^-1^ of sterile saline solution) to give two different nominal initial inoculum densities, namely 5×10^7^ (A1) and 2×10^8^ (A2) cfu g^-1^ dw of soil (freshly-spiked soil) or 8×10^7^ (B1) and 8×10^8^ (B2) cfu g^-1^ dw (aged soil); cell suspensions used as inoculum were prepared from *A*. *aurescens* TC1 late-exponential culture (OD_640_~1.6) grown in TC1 medium with 10 mM nitrogen from (NH_4_)_2_SO_4_. Upon soil bioaugmentation, moisture was adjusted to 60% soil water holding capacity (5.5 ml total added liquids per 100 g of soil dw) and soil microcosms were incubated in the dark at 25°C in a Thermostatic Cabinet (Lovibond ET618-4, Dortmund, Ge) during 14 days [13, 26]. Microcosms non-contaminated with the herbicide and not bioaugmented (designated as Ct-no TBA) or contaminated but not bioaugmented (designated as CT-no bacteria) were also included as controls. Triplicate microcosms were set up per treatment. All glassware and liquids used were sterilized by autoclaving or filtration.

Soil samples were collected immediately before (time zero) and after soil inoculation (at days 1, 3, 7, and 14) and processed for microbiological and chemical analysis, and ecotoxicity testing. To determine the concentration of total culturable bacteria (as cfu g^-1^ of soil dw) in the microcosms, soil samples (~1 g triplicates from each microcosm) were diluted in 10 ml of sterile 0.9% NaCl and 10-fold serial dilutions spread plated onto LB agar supplemented with cycloheximide (100 mg L^-1^) [26]. For chemical analysis, soil samples (approximately 5–10 g) taken from each triplicated microcosm were pooled and homogenized (total of ~20 g per treatment) and stored at -20°C until analysed. TBA and DET analysis was performed in ethylacetate extracts of soil samples by Gas Chromatography-Mass Spectrometry (limits of quantification of 0.010 mg kg^-1^ dw of soil), in the certified Laboratory of Chemical and Microbiological Analysis at the Instituto Superior Técnico (IST, UL, Lisbon, Portugal). For ecotoxicity testing, approximately 10 g of soil were collected from each replicated microcosm and stored at -20°C until use. Soil eluates and their toxicity testing with the model aquatic green microalgae *Pseudokirchneriella subcapitata* (strain Nr. WW 15–2521) were performed following standard protocols [27] and as described in detail elsewhere [13] to estimate algal growth as the specific growth rate (expressed as day^-1^).

### Statistical analysis

Three major questions were considered: (i) Was bacterial growth on ATZ, ammonium, urea or nitrate significantly different, and if yes was growth in the latter three sources better than in ATZ?; (ii) Was herbicide biodegradation (TBA or ATZ) by *A*. *aurescens* TC1 grown on the different nitrogen sources significantly different, and if yes was biodegradation rate in the latter three sources better than in ATZ?; and (iii) Was there decontamination of the TBA-contaminated (10×RD) soil microcosms upon bioaugmentation with *A*. *aurescens* TC1 inoculum grown in ammonium as sole nitrogen source, and if yes what was its efficacy? To answer question (i), *A*. *aurescens* TC1 specific growth rate and final cell density were tested through one-way analysis of variance (ANOVA), followed by the one-tailed Dunnett’s multiple comparison test to assess differences between growth with ATZ and each of the other nitrogen sources; effects of the nitrogen source concentration (2.8 or 10 mM) were tested by one-way ANOVA within the nitrogen sources ammonium or nitrate. For question (ii), differences in TBA or ATZ specific degradation rates by *A*. *aurescens* TC1 were evaluated by 2-way ANOVA to test for the effects of the main factors herbicide and nitrogen source, followed by planned comparisons to test for the effects of one main factor within the other and when necessary by the one-tailed Dunnett's multiple comparison test to assess biodegradation differences between cells grown in ATZ relatively to ammonium, urea or nitrate. As for question (iii), differences in the microalga specific growth rate were evaluated, separately for eluates from the fresh or the aged spiked soils, by two-way ANOVA to test for the main effects of inoculum (controls, A1 and A2, or controls, B1 and B2, respectively) and time (0, 1, 3, 7, and 14 days), followed by planned comparisons and Dunnett's test to explore the effects of inoculum within each time period; with 100% bioremediation efficacy for the combination of inoculum and time leading to algae growth equal to that in Ct-no TBA. The violations of normality and homoscedasticity were checked using Shapiro-Wilk’s and Bartlett’s tests, respectively, and statements of significant difference were set at the 0.05 level.

## Results

### Influence of nitrogen source on *Arthrobacter aurescens* TC1 inoculum growth

**Fig 1:**
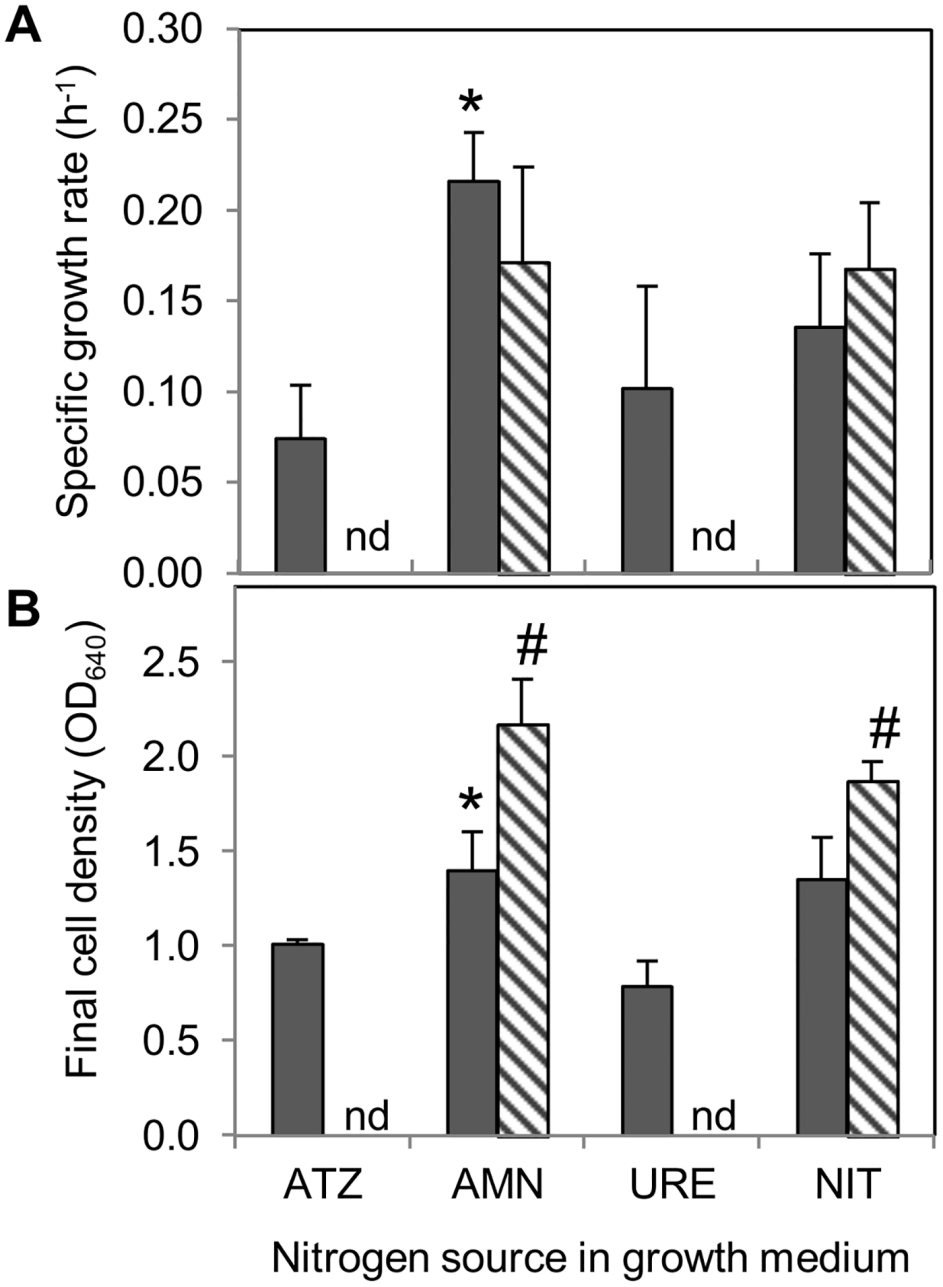
Influence of nitrogen source on *Arthrobacter aurescens* TC1 growth. Growth medium was supplemented with different nitrogen sources (ATZ—atrazine; AMN—ammonium; URE—urea; NIT—nitrate) at concentrations providing 2.8 mM (solid gray columns) or 10 mM (stripe pattern columns) nitrogen available for *A*. *aurescens* TC1. It is shown (A) the specific growth rate and (B) the final cell density (measured by OD_640_) attained in the stationary phase of growth in each medium. Error bars represent + 1 standard deviation. * indicates means significantly different from ATZ within each nitrogen concentration (one-tailed Dunnett's test). # indicates means on 2.8 and 10 mM nitrogen significantly different within each nitrogen source (one-way ANOVA; *p*-value < 0.05). nd indicates not determined.

The effects of growing the bacterium cultures in ammonium, urea or nitrate, compared with ATZ, as sole nitrogen sources, on the specific growth rate and the final cell density attained in the stationary phase of growth (after 10 h for growth on ammonium or 20–23 h for growth on ATZ, urea or nitrate), are shown in Fig 1. *Arthrobacter aurescens* TC1 grew well using 2.8 mM nitrogen supplied from any of the four nitrogen sources (Fig 1). Yet, the bacterium specific growth rate (Fig 1A) and final cell density (Fig 1B) in ammonium were significantly higher than when grown in ATZ (*p* = 0.0083 and *p* = 0.048, respectively) and also in urea (*p* = 0.027 and *p* = 0.0086, respectively), but similar to those in nitrate (*p* = 0.11 and *p* = 0.96, respectively) (Fig 1). On the other hand, the presence of ammonium or nitrate at the highest concentration of 10 mM nitrogen did not significantly modify the bacterium specific growth rate (*p* = 0.16 or *p* = 0.36, respectively) (Fig 1A), even though the final cell density was significantly higher with 10 mM compared with 2.8 mM nitrogen for both ammonium (*p* = 0.0039) and nitrate (*p* = 0.021) (by 55% or 39%, respectively) (Fig 1B).

### Influence of nitrogen source on *A*. *aurescens* TC1 herbicide biodegradation rate

**Fig 2:**
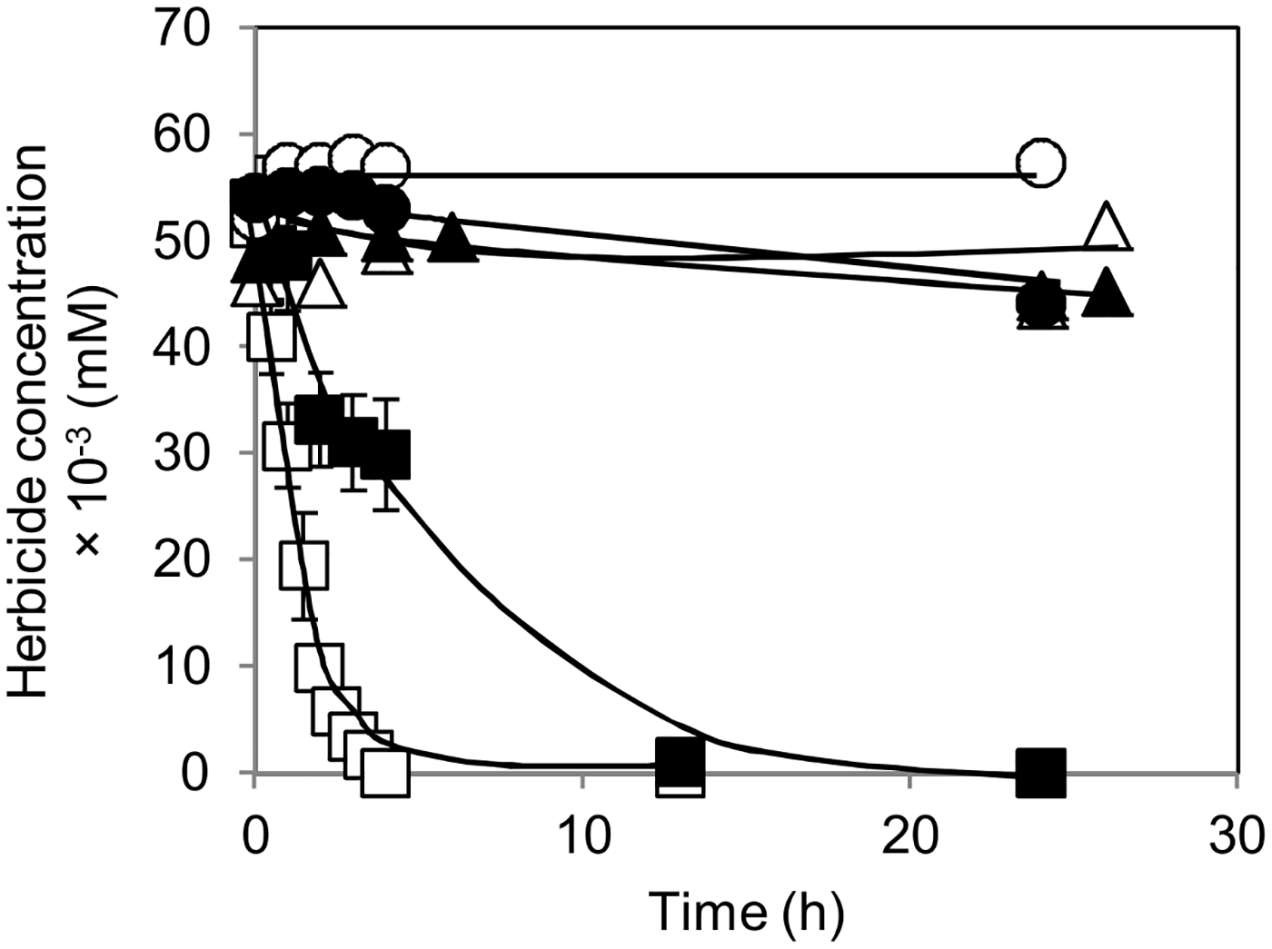
Herbicide biodegradation by ammonium-grown *Arthrobacter aurescens* TC1. The bacterium cells were grown in medium with 2.8 mM nitrogen from ammonium. It is represented the time-course (in hours, h) variation curves of terbuthylazine (■, ●, ▲) or atrazine (□, ○, △) concentration in the supernatant of phosphate-salt buffer (pH 7) supplemented with each herbicide (initial concentration ~ 0.05 mM) and inoculated at time zero with the bacterium cells (■, □) or with cells killed by boiling (▲, △), or non-inoculated (●, ○). Error bars represent ± 1 standard deviation.

To further examine whether *A*. *aurescens* TC1 cells grown in the different nitrogen sources were effective in the degradation of chlorinated *s*-triazine herbicides, their ability to remove TBA (or ATZ, for comparison purposes) from a phosphate-salt buffer (pH 7.0) was examined. The time-course herbicide degradation curves for cells grown in ammonium are shown in Fig 2, as an example of the degradation curves obtained.

**Fig 3:**
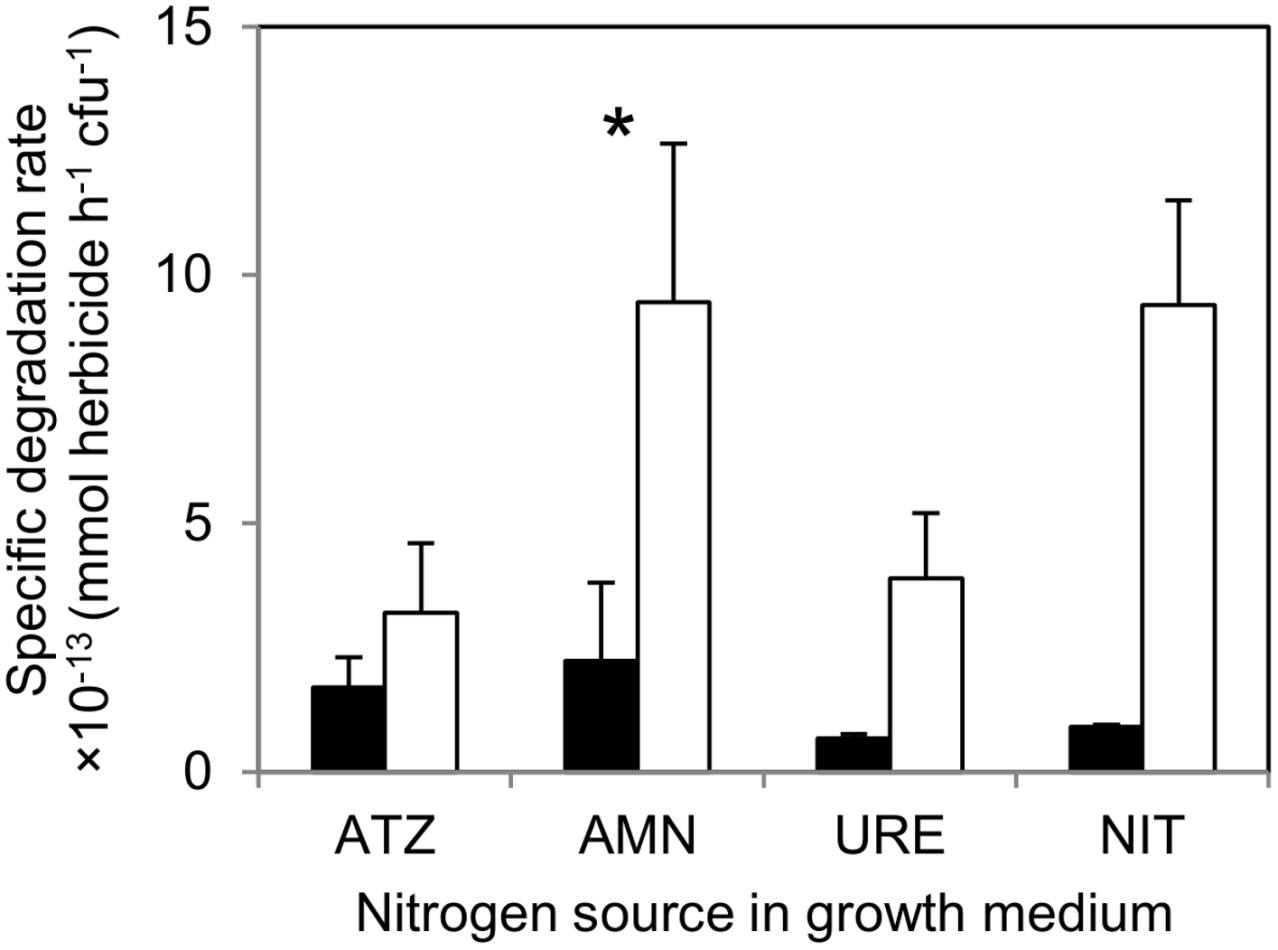
Influence of the nitrogen source for growth on *Arthrobacter aurescens* TC1 herbicide biodegradation rate. Specific terbuthylazine (black bars) or atrazine (empty bars) degradation rate values determined in phosphate-salt buffer (pH 7.0 ± 0.2; initial herbicide concentration ~ 0.05 mM) with bacterium cells grown in media containing 2.8 mM nitrogen from different nitrogen sources (ATZ—atrazine; AMN—ammonium; URE—urea; NIT—nitrate). Error bars represent + 1 standard deviation.* indicates means significantly different from ATZ as nitrogen source (by one-tailed Dunnett's test) irrespectively from the herbicide biodegraded because the interaction effect between the two main factors was not significant.

In general, assays with cells grown in ammonium, urea, nitrate or ATZ (all providing approximately 2.8 mM nitrogen) showed clearly a reduction of the initial TBA or ATZ concentration in the supernatant, contrary to controls without cells or with cells killed by boiling (Fig 2, and data not shown). Even though TBA removal from the supernatants was generally slower than that of ATZ, the concentrations of both herbicides were undetectable after 13 or 5 h of incubation, respectively, indicating that cells exhibited complete herbicide removal (Fig 2, and data not shown). Based on the biodegradation curves obtained with the bacterial cells grown on each nitrogen source (Fig 2, and data not shown), the respective TBA or ATZ specific degradation rates were calculated (S1 Table) and compared in Fig 3.

Two-way ANOVA results showed specific herbicide degradation rate to be significantly affected by the nitrogen source (F_3,14_ = 3.49, *p* = 0.044) and the herbicide (F_1,14_ = 28.0, *p* < 0.00011) but not by the significant interaction effect (F_3,14_ = 2.15, *p* = 0.14). Overall, TBA specific degradation rates were significantly lower than the ATZ ones (overall means of 1.7 × 10^−13^ or 7.6 × 10^−13^ mmol herbicide h^-1^ cfu^-1^, respectively), independently from the nitrogen source used for growth (Fig 3). On the other hand, ammonium-grown cells showed significantly higher herbicide degradation rate values compared with ATZ-grown cells (*p* = 0.044; overall means of 6.0 × 10^−13^ or 3.0 × 10^−13^ mmol herbicide h^-1^ cfu^-1^, respectively) (Fig 3). Similarly, cells of *A*. *aurescens* TC1 cultured in 10 mM nitrogen from ammonium were effective in TBA (or ATZ) removal from the phosphate-salt buffer (data not shown). Based on obtained results, the further examination of the efficacy of *A*. *aurescens* TC1 as a bioaugmentation tool, in soil microcosms contaminated with TBA, was conducted with bacterium inoculum cells grown in TC1 medium supplemented with 10 mM nitrogen from ammonium.

### Efficacy of TBA biodegradation in soil microcosms

**Fig 4:**
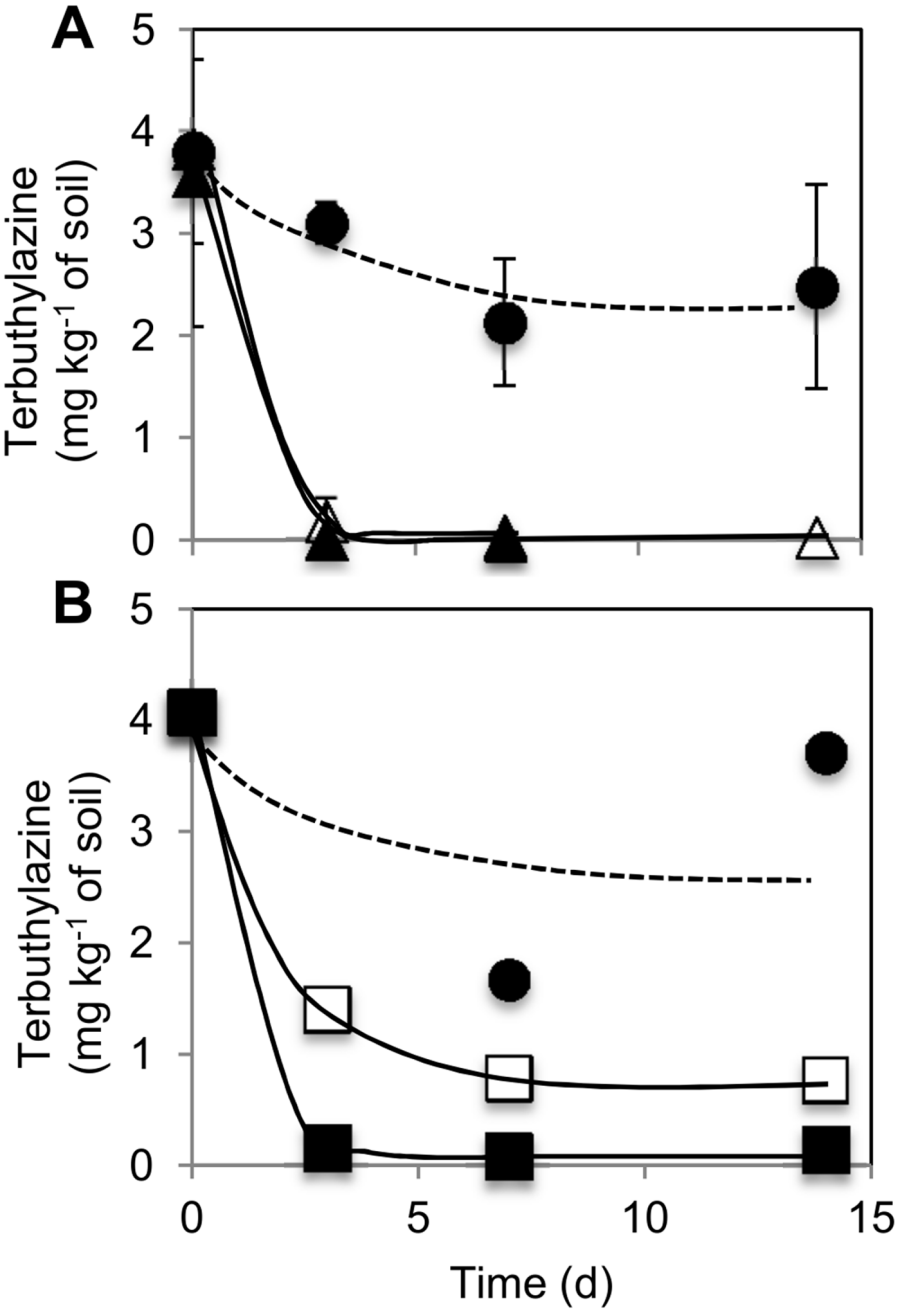
Terbuthylazine removal from soil microcosms upon bioaugmentation with ammonium-grown *Arthrobacter aurescens* TC1 inocula. Time-course (in days, d) variation of terbuthylazine concentration in soil microcosms contaminated with (A) fresh or (B) four month-aged Terbutilazina-Sapec (both at 10× the recommended field dose for weed control in corn cultivation) and bioaugmented (at day zero) with viable cells of *A*. *aurescens* TC1 at the following initial inoculum densities: 5 × 10^7^ (△), 8 × 10^7^ (□), 2 × 10^8^ (▲), or 8 × 10^8^ (■) cfu g^-1^ dry weight of soil. Terbuthylazine concentration measured in the non-bioaugmented soil is also shown (●). Error bars represent ± 1 standard deviation.

TBA removal from soil microcosms as a result of soil bioaugmentation with ammonium-grown *A*. *aurescens* TC1 was examined in soil freshly- or aged-spiked with a TBA commercial formulation at 10×RD, to represent worst-case situations of accidental herbicide contamination of soil. In each case, the influence of two different *A*. *aurescens* TC1 initial inoculum densities was addressed and results are presented in Fig 4.

In the freshly spiked soil, the addition of both 5 × 10^7^ and 2 × 10^8^ cfu of *A*. *aurescens* TC1 g^-1^ dw of soil resulted in the rapid removal, during the first 3 days, of most initial TBA (> 95%; from 3.8±1.7 to less than 0.2 mg TBA kg^-1^ dw of soil) (Fig 4A). On the contrary, a high TBA residual concentration of 2.7 ± 1.0 mg TBA kg^-1^ dw of soil (i.e., around 70% in average of initial TBA) remained in the non-bioaugmented control soil during the 14 days bioremediation period (Fig 4A). Comparatively, TBA aging in soil led to a decreased rate and extent of TBA biodegradation, particularly for the soil bioaugmented with the inoculum density of 8 × 10^7^ cfu of *A*. *aurescens* TC1 g^-1^ dw of soil), with residual TBA always higher than 0.8 mg kg^-1^ of soil up to day 14 (Fig 4B). Nevertheless, a 10-fold higher inoculum density allowed almost complete removal of aged TBA, which declined to less than 0.2 mg kg^-1^ dw of soil already at day 3 (Fig 4B). It should be noted that during the course of the bioaugmentation experiments in soil microcosms with either fresh or aged TBA the chlorinated metabolite DET did not accumulate; its levels were always below 0.1 mg kg^-1^ dw of soil. Regarding the fate of *A*. *aurescens* TC1 cells in soil during the bioremediation experiments, some information was obtained from the numbers of total culturable bacteria counted (using LB medium) in the bioaugmented compared with the non-bioaugmented soil microcosms (data in S1 File). These comparisons indicated the presence of viable cells of the bioaugmentation bacterium in soil after completion of TBA removal (> 3 days, Fig 4A and 4B) but a decline in their numbers from there onwards (S1 File).

**Fig 5:**
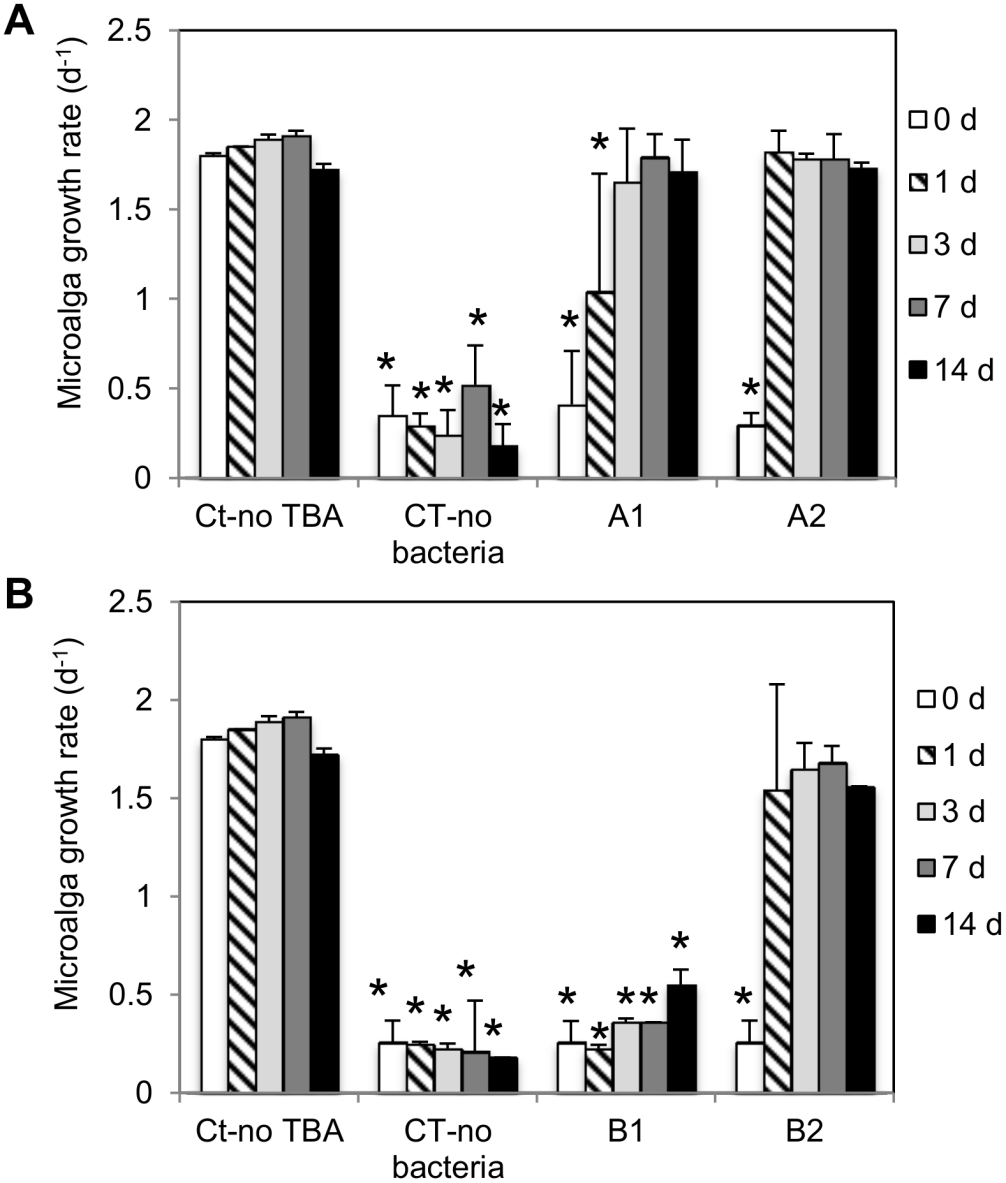
Ecotoxicity towards a microalga of eluates prepared from bioaugmented or non-bioaugmented soil microcosms. The mean 72-h growth rate of *Pseudokirchneriella subcapitata* was determined in eluates prepared from soil collected at the indicated time periods (in days, d) from the microcosms contaminated with (A) fresh or (B) four month-aged Terbutilazina-Sapec (at 10× the recommended dose for weed control in corn cultivation) and subsequently bioaugmented (at day zero) with viable cells of *Arthrobacter aurescens* TC1 (added viable cells g^-1^ dry weight of soil, as follows: 5×10^7^-A1, 2×10^8^-A2, 8×10^7^-B1, 8×10^8^-B2). Ecotoxicity of eluates from soils microcosms non-contaminated with the herbicide (Ct-no TBA) or spiked but non-bioaugmented (CT-no bacteria) are also shown. Error bars represent + 1 standard deviation. * indicates means significantly different from clean soil (CT-no TBA) within each time (by Dunnett’s test).

The efficacy of the bioremediation process and its potential to minimize environmental contamination via the soil-water pathway was further examined through the ecotoxicity testing (using a microalga) of eluates prepared from the soil samples collected in the freshly-spiked (Fig 5A) and the aged-spiked microcosms (Fig 5B) throughout time (from 0 up to 14 days). Two-way ANOVA results showed that time and inoculum density as well as their interaction significantly influenced microalga growth, for both the freshly (F_4,59_ = 27.4, *p* < 0.001; F_3,59_ = 186, *p* < 0.001 and F_12,59_ = 13.4, *p* < 0.001, respectively) and the aged-spiked soils (F_4,22_ = 13.1, *p* < 0.001; F_3,22_ = 294, *p* < 0.001 and F_12,22_ = 11.4, *p* < 0.001, respectively). Eluates from the TBA-contaminated soil non-bioaugmented with *A*. *aurescens* TC1 (CT-no bacteria) significantly inhibited microalga growth at all timings in both spiking scenarios by 73 to 90% (*p* < 0.001), proving 10×RD of TBA to be highly toxic for the microalga (Fig 5A and 5B). In the aged TBA microcosms bioaugmented with the lowest inoculum density (B1, in Fig 5B), there was no significant decrease in the ecotoxicity of soil eluates and thus no effective bioremediation occurred up to 14 days (Fig 5B). Yet, remarkably, soil eluates from the microcosms spiked either with fresh TBA and bioaugmented with both inoculum densities (A1 and A2, in Fig 5A) or with aged TBA and bioaugmented with the highest inoculum density tested (B2, Fig 5B) were, after 3 days, no longer toxic for the microalga (*p* > 0.40) (Fig 5). Moreover, microalga growth in these eluates differed by 0 to merely 13% from the growth in eluates from clean soil without herbicide (Ct-no TBA), indicating a 100% bioremediation efficacy (Fig 5).

## Discussion

In the present work, we first demonstrated the feasibility of growing *A*. *aurescens* TC1 inocula for TBA-contaminated soils bioremediation in three simple sole nitrogen sources (namely, ammonium, urea or nitrate) instead of ATZ. It would be reasonable to anticipate bacterial cultivation without the *s*-triazine selective pressure (i.e., without ATZ) to trigger catabolic plasmid instability and thus impair cells performance with respect to chloro-*s*-triazine herbicide biodegradation [20, 23]. The influence of the nitrogen source for growth on the stability of the *A*. *aurescens* pTC1 plasmid (harbours the *s*-triazine catabolic genes *trzN*, *atzB* and *atzC* [20]) or in the expression of these catabolic genes is not addressed in the present work. Nevertheless, it seems relevant in this context that, independently of the nitrogen source used for *A*. *aurescens* TC1 growth, the relative values of specific TBA or ATZ degradation rates measured *in vivo* (i.e., about 4.5-times lower for TBA degradation than for ATZ’s, in phosphate buffer) are consistent with the 5-times lower activity of the *A*. *aurescens* TC1 purified TrzN enzyme with TBA as substrate compared to ATZ [21]. More importantly, the fact that bacterial cultures grown in ATZ absence are still able to efficiently remove TBA (or ATZ) from the phosphate buffer or the soil microcosms has important practical advantages. In this way, the use of ATZ as nitrogen source in the bacterium growth medium can be avoided, with cost-effectiveness and environment-friendly advantages (e.g., less toxic residues to be disposed in the inoculum preparation). We anticipate this aspect is a major contribution to improve the applicability of the bioaugmentation tool in the field.

Obtained results also point out TBA or ATZ biodegradation by *A*. *aurescens* TC1 to be not inhibited by nitrogen sources supporting significantly faster bacterial growth, such as ammonium or nitrate compared with ATZ-growing cells. This observation is consistent with the known bacterium ability to metabolize the *N*-alkyl amines liberated from TBA or ATZ *s*-triazine ring as well as a wide diversity of environmental nitrogen compounds besides. [21]. Likewise, other soil *Arthrobacter* and *Agrobacterium* strains for which ATZ-biodegradation was found not to be under limitation of preferential inorganic nitrogen sources in the growth medium have been reported [20, 24, 28, 29]. On contrary, the nitrogen repression of ATZ-biodegradation is well-known in *Pseudomonas* sp. ADP and other soil bacteria, contributing to restrict the uses of these bacterial strains in the bioremediation of chloro-*s*-triazine herbicide pollution in agricultural soils, which often contain added nitrogen fertilizers [20, 24, 25]. In the present work, the fact that the nitrogen sources most favorable for growth, ammonium and nitrate, did not hinder *A*. *aurescens* TC1 ability to biodegrade TBA (or ATZ) points to a major practical advantage associated to the use of this bacterium as bioaugmentation tool to remediate high TBA contamination for instance in the event of spill situations in land. Indeed, accidentally released chemical mixtures may contain diverse nitrogen compounds, including chemical fertilizers, besides pesticides [11, 30].

In the sequence, experimental evidences provided in the present work showed the remarkable bioremediation efficacy of ammonium-grown *A*. *aurescens* TC1 cells as bioaugmentation tool, in bench-scale soil microcosms freshly- or aged-spiked with Terbutilazina-Sapec at an extreme dose (10 × the recommended field dose) to represent spill situations. Indeed, soil bioaugmentation with the *A*. *aurescens* TC1 inocula accelerated TBA removal from the soil microcosms. The observed decline in TBA residual concentration in soil to considerably less than the predicted environmental concentration associated to a single application of the commercial formulation at the recommended field rate (i.e., 1 mg kg^-1^ dw of soil [1]) is considered an adequate level of herbicide removal from the contaminated soils [1, 16]. More environmentally relevant seems the abatement of soil eluates ecotoxicity towards a standard freshwater microalga observed upon 3 days of the bioaugmentation treatments; these results pointed to the eventual complete decontamination of the soil and thus a bioremediation efficacy of 100%. On contrary, the ecotoxicity assessment of eluates obtained from the non-bioaugmented microcosms confirmed herbicide mobilization via the soil water pathway (e.g., due to leaching and/or runoff events) [1, 5, 12] does pose threats to primary producers in aquatic systems, as reported by others [7, 31]. In the present work, overall results thus suggest the environmental risk of TBA associated to the mobilization of the herbicide to aquatic ecosystems, in the event of a spill situation, can be potentially reduced or prevented by using this bioaugmentation tool, as reported before with respect to the bioremediation of ATZ-contaminated soils using *Pseudomonas* sp. ADP [13, 14]. The first hydrolytic displacement of the chlorine substituent from the *s*-triazine ring of TBA to produce hydroxyterbuthylazine and its further transformation into the end-product cyanuric acid performed by the bioaugmentation bacterium *A*. *aurescens* TC1 [20] eventually contribute for soil decontamination as highlighted in the present work. Nevertheless, it is reasonable to expect the cyanuric acid formed from TBA by *A*. *aurescens* TC1to be further metabolized by soil indigenous microorganisms, as for instance in the case of the soil microcosms herein used (data in S2 File). As a matter of fact, cyanuric acid-hydrolysing enzymes and -metabolising microorganisms have been reported before to be common within the soil ecosystem [32].

The results that were obtained in the present work also indicated the inoculum density of the bioaugmentation bacterium to be an important factor influencing the time needed to achieve effective soil decontamination, particularly in the less favorable case of herbicide aging in soil. This latter situation is relevant to represent real cases of soil contamination associated with, for instance, prolonged accidental spills or deficient storage/operation in dealerships and mix-load sites or inadequate disposal [11, 16, 18, 30]. The apparent lower effectiveness of *A*. *aurescens* TC1 to remove the aged TBA compared with the freshly-spiked herbicide herein observed may be related with the lowering of TBA bioavailability during aging [33] due to its favorable sorption to soil organic carbon (*K*_OC_ = 231 L kg^-1^ [2]) associated with its hydrophobic nature (Log P = 3.4 [2]). Aging of TBA (or ATZ) in soil and aquifer sediment was also reported before to result in reduced rate and degree of mineralization of *s*-triazine herbicides by *Pseudomonas* sp. ADP and attributed mainly to reduced herbicide bioavailability [18]. In practical terms, the fact that relatively high numbers of viable cells in the bioaugmentation inoculum (namely, 8 × 10^8^ or 5 × 10^7^ per g of soil for aged- or freshly-spiked soils, respectively) may be needed to achieve successful soil decontamination in the experimental conditions herein used may be seen as a drawback for the use of this bioaugmentation tool in the field. Nevertheless, we anticipate it as worthwhile in the event of high contamination due to accidental spills or persistent pollution in soils that contain low numbers of, or poor, TBA degraders. It is, for instance, the case of the natural soil microcosms used in the present work, which do not support intrinsic biodegradation of ATZ [26] or TBA (data in S2 File).

In conclusion, even though the practicability of the herein examined bioaugmentation tool in TBA-contaminated soils at larger scales and in field situations still requires optimization on a case-by-case basis, the present study highlights its efficacy at rapidly reducing the potential environmental risks of TBA in the event of high soil contamination, contributing to minimize aquatic ecosystems impacts. Particularly, the bacteria initial inoculum density and the herbicide aging in soil are pointed out as relevant factors influencing the time needed to achieve effective soil decontamination. Other important factors might be the type of soil, the level of soil contamination and diverse environmental conditions that may vary seasonally [34]. Further studies are also needed to address the influence of cells formulation and conservation methods as well as of storage conditions and time in the performance and efficacy of *A*. *aurescens* TC1 cultures as bioaugmentation system, which are underway.

## Supporting Information

S1 File Total culturable bacteria units in the soil microcosms during the bioremediation experiments. (PDF)

S2 File Mineralization experiments with [*UL*-ring-^14^C]terbuthylazine. (PDF)

S1 Table Values of specific TBA or ATZ degradation rates (expressed as mmol herbicide h^-1^ cell^-1^) by *A*. *aurescens* TC1 cultures grown with different sole nitrogen sources. (XLSX)
