# Supplementary material for: Evaluation of Arthrobacter aurescens Strain TC1 as Bioaugmentation Bacterium in Soils Contaminated with the Herbicidal Substance Terbuthylazine
Source: PLoS One. 2015 Dec 14;10(12):e0144978. doi: 10.1371/journal.pone.0144978 (PMC4684385; doi:10.1371/journal.pone.0144978)
Supplement: S1 File — (PDF) [file pone.0144978.s001.pdf]

S1 File. Total culturable bacteria units in the soil microcosms during the bioremediation experiments

Data of total colony forming units (cfu) counted in the non-bioaugmented soil indicate the soil microcosms support considerable numbers of indigenous bacteria (1.6±0.6 $\times 10^{8}$ total cfu g^{-1} of soil, in average) (Figure below). Comparisons of total cfu numbers between the bioaugmented and the non-bioaugmented soil microcosms indicate the presence of viable cells of the bioaugmentation bacterium during up to 14 days of the bioremediation treatments but a decline in their numbers from day one/three onwards (Figure below).

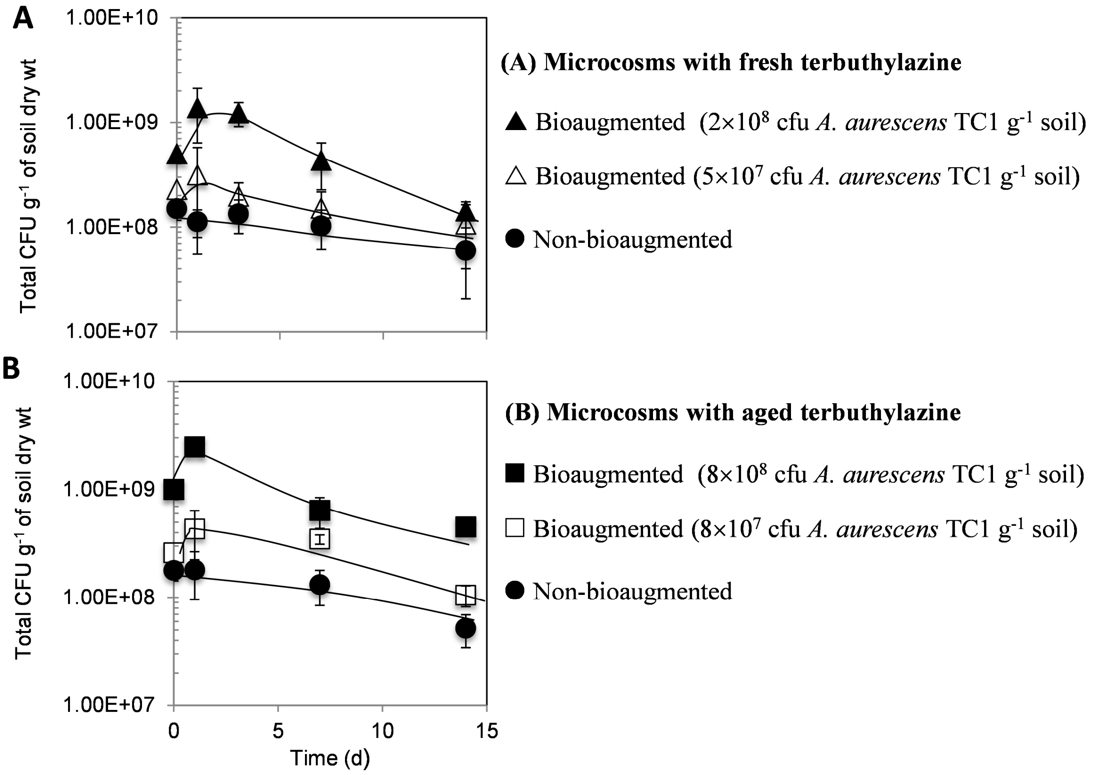

Fig. Total culturable bacteria units in the soil microcosms during the bioremediation experiments. Time-course (in days, d) variation of total colony forming units (cfu) counted (using agarized LB medium) in the soil microcosms contaminated with (A) fresh or (B) four month-aged Terbutilazina-Sapec (10× the recommended field dose) and bioaugmented (at day zero) with viable cells of *A. aurescens* TC1 at the indicated initial inoculum densities. Total cfu g^{-1} of soil counted in the non-bioaugmented soil is also shown for comparison. Error bars represent ± 1 standard deviation.
