# Supplementary material for: Evaluation of Arthrobacter aurescens Strain TC1 as Bioaugmentation Bacterium in Soils Contaminated with the Herbicidal Substance Terbuthylazine
Source: PLoS One. 2015 Dec 14;10(12):e0144978. doi: 10.1371/journal.pone.0144978 (PMC4684385; doi:10.1371/journal.pone.0144978)
Supplement: S2 File — (PDF) [file pone.0144978.s002.pdf]

S2 File. Mineralization experiments with [*UL*-ring-^{14}C]terbuthylazine

The terbuthylazine (TBA)-catabolic pathway of the bioaugmentation bacterium *Arthrobacter aurescens* strain TC1 funnels into cyanuric acid, which accumulates stoichiometrically (1:1) being no further mineralized [19, 20]. Cells of *A. aurescens* TC1 are thus not expected to release $^{14}CO_2$ from the [*UL*-ring-^{14}C]TBA molecule (i.e., with the *s*-triazine ring carbons labelled with ^{14}C) [19]. Screening of the natural agricultural soil used in the present work for total culturable bacteria showed this soil to support considerable numbers of indigenous microorganisms (1.6±0.6 ×10^8 total cfu g^{-1} of soil in average) (S1 File). In this context, the present additional experiment aimed at getting clues regarding the possible role of the soil indigenous microorganisms on the fate of the cyanuric acid formed from TBA biodegradation by *A. aurescens* TC1 in the bioaugmented soil microcosms. To do that, we examined whether the ^{14}C-labelled *s*-triazine ring of cyanuric acid formed from [*UL*-ring-^{14}C]TBA by *A. aurescens* TC1 in the bioaugmented soil could be further mineralized to $^{14}CO_2$ by the soil indigenous microbial populations. Mineralization assays were carried out in both fresh and sterilized natural soil at 25±0.3 ºC, as described elsewhere [26]; soil was sterilized by autoclaving on three consecutive days (confirmed to have no culturable microbial populations by direct spread plating onto LB medium). In both cases, soil was spiked with a mixture of [*UL*-ring-^{14}C]TBA (99.9% purity, specific activity 1.17 GBq $mmol^{-1}$, IZOTOP, Budapest, Hu) and non-labelled TBA from Terbutilazina – Sapec, at a total approximate concentration of 10 mg TBA kg^{-1} of soil (as in main text). TBA-spiked soils were then bioaugmented with a suspension of ammonium-grown *A. aurescens* TC1 cells (at time zero; initial inoculum density ~3 × 10^8 cfu g^{-1} of soil) or not inoculated. The time-course evolution of $^{14}CO_2$ released during the subsequent 30-days incubation period is shown in the figure below.

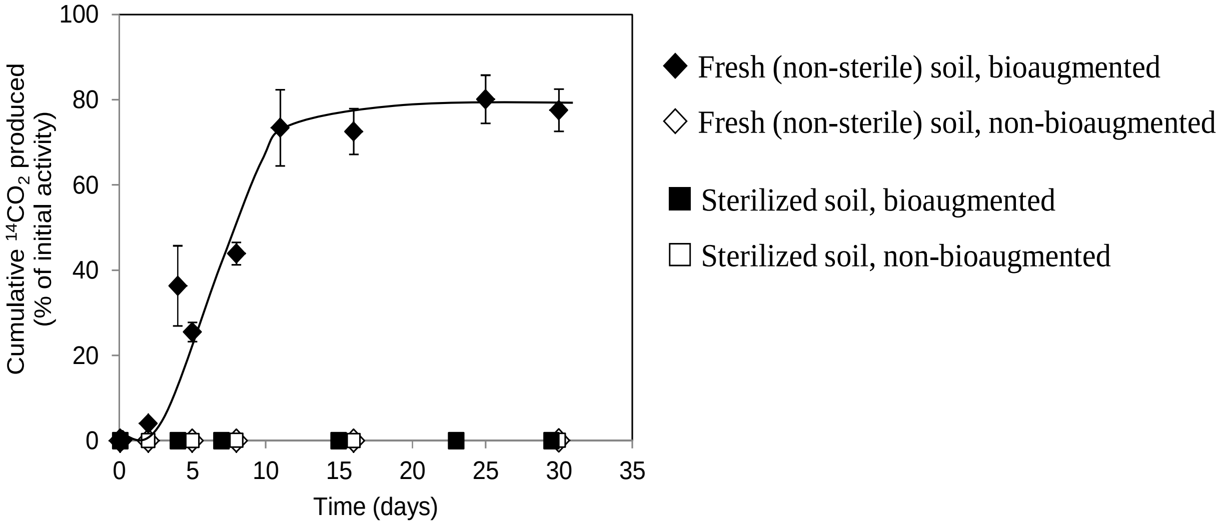

Fig. Mineralization experiments with [*UL*-ring-^{14}C]terbuthylazine. It is represented the time-course evolution of $^{14}CO_2$ released from non-sterile or sterilized soil freshly-spiked with [*UL*-ring-^{14}C]terbuthylazine, and bioaugmented with *Arthrobacter aurescens* TC1 (~3×10^{8} colony forming units g^{-1} of soil, at time zero) or non-bioaugmented. Error bars represent ± 1 standard deviation.

In the non-sterile non-bioaugmented soil, no $^{14}CO_2$ accumulation occurred during at least 30 days (Figure above). The presence in this soil of indigenous microorganisms not capable of mineralizing the *s*-triazine ring of TBA is not surprising, since the soil used has no history of prior herbicide exposure and it was shown before to support no effective intrinsic mineralization of the *s*-triazine ring of atrazine [26]. Also, this result is consistent with the observation that in the non-bioaugmented soil microcosms of the bioremediation experiments almost all initial TBA (>70% of initial) remained in the soil during at least 15 days (main text). Similarly, in the sterilized soil bioaugmented with the *A. aurescens* TC1 inoculum, no $^{14}CO_2$ evolved from the ring-labelled TBA (Figure above), as expected [19, 20]. Comparison of the latter with the data obtained in the fresh (non-sterile) soil bioaugmented with *A. aurescens* TC1 cells (approximately 80% of initial ring-labelled TBA mineralized to $^{14}CO_2$ after 15 days) (Figure above), suggests $^{14}CO_2$ formation under these conditions may be due to the

activity of the soil indigenous microbial populations. Altogether, results point to the existence of indigenous microorganisms in the natural soil used in the present work able to carry out the mineralization of the cyanuric acid previously formed from TBA transformation by *A. aurescens* TC1.

Note: the reference numbers herein used are the ones of the references listed in the main text.
